# The Incorporation of Chitosan in the Antibacterial Capability and Biocompatibility of a Protein-Repellent Orthodontic Cement

**DOI:** 10.7759/cureus.66099

**Published:** 2024-08-03

**Authors:** Jianru Yi, Fan He, Zhihe Zhao

**Affiliations:** 1 Department of Orthodontics, State Key Laboratory of Oral Diseases, National Clinical Research Center for Oral Diseases, West China School of Stomatology, Sichuan University, Chengdu, CHN

**Keywords:** biocompatibility, mpc, chitosan, orthodontic cement, white spot lesions

## Abstract

Background: This study aimed to develop an orthodontic cement containing chitosan and 2-methacryloyloxyethyl phosphorylcholine (MPC) and to investigate its antibacterial properties and biocompatibility.

Methods: Chitosan and MPC were incorporated into commercial cement. The enamel bonding strength and biocompatibility of the new cement were evaluated. The antibacterial properties were assessed by examining biofilm metabolic activity and colony-forming units (CFU). An evaluation of the protein repellency of the cement was also conducted.

Results:* *The new cement containing chitosan and MPC had clinically acceptable bonding strength. In comparison to the control, the novel cement demonstrated enhanced protein-repellent properties (p < 0.05), inhibited biofilm metabolic activity (p < 0.05), and reduced CFU counts (p < 0.05) without diminishing cell viability in response to cement extracts (p > 0.05).

Conclusions: The synergistic application of chitosan and MPC endows the cement with potent antibacterial abilities, protein repellency, and favorable biocompatibility.

## Introduction

The demand for orthodontic treatments has witnessed significant growth due to an increased emphasis on quality of life and aesthetic preferences [1]. Nonetheless, fixed orthodontic devices introduce complexities in oral hygiene maintenance and are prone to bacterial colonization. A notable adverse effect of fixed orthodontic treatments is the formation of white spot lesions (WSLs) [2], which not only detract from aesthetic appeal and patient contentment but also indicate underlying enamel damage [3]. To address this, the development of effective WSL prevention methods has become imperative.

Orthodontic cement has emerged as a promising candidate for WSL prevention. Its effectiveness, independent of patient adherence and its strategic application in areas susceptible to WSLs, renders it a compelling choice. Researchers have extensively explored cement modifications to combat WSLs [4,5]. A notable advancement is the integration of 2-methacryloyloxyethyl phosphorylcholine (MPC) into dental materials. MPC contains zwitterionic phosphorylcholine groups, which mimic cell membrane structures. Its unique surface structure prevents nonspecific protein adsorption [6]. Recent studies have successfully integrated MPC into dental materials to reduce bacterial attachment [7-9].

Chitosan, a linear polysaccharide derived from the exoskeletons of arthropods such as crustaceans, has garnered significant attention in the biomedical field due to its remarkable biocompatibility, biodegradability, and inherent antibacterial properties [10]. This biopolymer is composed of randomly distributed β-(1→4)-linked D-glucosamine and N-acetyl-D-glucosamine units, which contribute to its unique physicochemical characteristics [11]. In dental materials, chitosan has been extensively studied for its potential to enhance antibacterial efficacy, thereby reducing the incidence of dental caries and periodontal diseases. The incorporation of chitosan into dental composites, sealants, and adhesives has demonstrated a substantial reduction in bacterial colonization and biofilm formation [11,12]. Hence, it holds promise for augmenting the antibacterial activity of orthodontic cement in conjunction with other biomaterials, which has not been investigated yet.

This study aimed to innovate in orthodontic cement development by integrating MPC and chitosan into a resin-modified glass ionomer (RMGI) for the first time. We examined the following hypotheses: (1) The bonding strength of the new cement is comparable to that of commercial RMGI; (2) the addition of MPC and chitosan enhances the antibacterial capability and protein-repellent ability of RMGI; and (3) the incorporation of MPC and chitosan does not compromise the biocompatibility of RMGI.

## Materials and methods

Incorporation of MPC and chitosan into RMGI

The protocol of this study was approved by the Research Ethics Committee of the author’s affiliation. This study utilized a commercially available RMGI (GC Ortho LC, Fuji, Japan), henceforth referred to as GC, as the base system. GC is conventionally employed in orthodontic practices for bracket bonding. According to the manufacturer’s instructions, GC is composed of fluoroaluminosilicate glass, hydroxyethyl methacrylate, and a photoreactive acid, with a recommended powder-to-liquid mass ratio of 2.5. MPC (Sigma-Aldrich, Mo., USA) was integrated into the cement at a mass fraction of 2%, according to our previous study [13]. Chitosan (Sigma-Aldrich) was initially introduced to the GC liquid, which was then subjected to sonication and vortexing for one hour to ensure homogeneity [14]. Subsequently, the GC powder was mixed with the chitosan-infused liquid at a mass ratio of 2.5. Three cements with chitosan mass fractions of 0.3%, 0.4%, and 0.5% were prepared. A mass fraction exceeding 0.5% was not involved due to the resultant compromised bonding strength.

Shear bonding strength test

For the shear bonding strength (SBS) assessment, 36 human first premolars, extracted with informed consent for orthodontic reasons, were vertically embedded in self-curing acrylic resin (Fuji, Japan). The enamel surface underwent a 30-second etching process using 37% phosphoric acid gel (XihuBiom, Hangzhou, China), after which metal brackets (XihuBiom, Hangzhou, China) were bonded to the buccal surface center of each tooth using the specified cements. The tested groups included:

(1) GC control, (2) GC + 2% MPC (GC+MPC), (3) GC + 0.3% chitosan (GC+0.3% chitosan), (4) GC + 0.4% chitosan (GC+0.4% chitosan), (5) GC + 0.5% chitosan (GC+0.5% chitosan), and (6) GC + 2% MPC + 0.4% chitosan (GC+MPC+0.4% chitosan).

The SBS was quantitatively determined. A chisel linked to a universal testing machine was positioned above the bracket base to exert a mechanical force. The peak load was recorded when bracket detachment occurred. The SBS was computed by dividing the peak load by the surface area of the bracket base [5,13].

Specimen preparation

Subsequent to the SBS evaluation, four groups were included in the subsequent experiments: GC control, GC+MPC, GC+0.4% chitosan (referred to as GC+chitosan), and GC+2%MPC+0.4% chitosan (GC+MPC+chitosan). Cement pastes were molded into a circular form with a diameter of 8 mm and an approximate thickness of 0.6 mm, followed by a one-minute photopolymerization. The cured disks were then immersed in deionized water and stirred by a magnetic bar at a rate of 100 rpm for 1 hour to eliminate unreacted monomers [15].

Biofilm formation

*Streptococcus mutans* (ATCC 700610), the primary etiological agent of dental caries, was cultured to develop biofilms for the evaluation of antibacterial efficacy. An overnight anaerobic incubation at 37°C in brain-heart infusion (BHI) broth (BD, NJ, USA) was conducted with a *Streptococcus mutans* stock. The inoculum was standardized to approximately 107 colony-forming units/mL. Cement disks were arrayed in a 24-well plate and submerged in 1.5 mL of the inoculum. Following 24 hours of incubation at 37°C with a 5% CO2 condition, the disks were transferred to a new 24-well plate with fresh BHI and incubated for an additional 24 hours to promote mature biofilm formation on the cement surfaces [16].

Antibacterial efficacy evaluation

The antibacterial attributes were appraised using the 3-(4,5-dimethyl-thiazol-2-yl)-2,5-diphenyltetrazolium bromide (MTT) metabolic assay and colony-forming unit (CFU) assessments [15,17]. For the MTT assay, cement disks harboring two-day biofilms were placed in new 24-well plates and covered with 1 mL of MTT solution for a one-hour incubation. The disks were then transferred to new plates and submerged in 1 mL of dimethyl sulfoxide (DMSO). After a 20-minute incubation in darkness, the optical density of the DMSO solution at 540 nm was recorded. For CFU tests, biofilm-coated disks were transferred into tubes containing 2 mL of cysteine peptone water. Biofilm was harvested through sonication and vortexing, serially diluted, and then plated onto BHI agar. Colony counts were performed after a 48-hour incubation.

Protein-repellent ability test

The cement disks of each group were immersed in a 4.5 g/L bovine serum albumin (BSA, Sigma-Aldrich) solution at 37°C for two hours. Subsequently, the disks were immersed in 1% sodium dodecylsulfate (SDS) in PBS and sonicated for 20 minutes to collect the BSA adsorbed onto the cement surface. The concentration of BSA within the SDS solution was determined using a commercial protein analysis kit (micro BCA protein assay kit, Fisher Scientific, PA, USA).

Biocompatibility analysis

The cement disks were submerged in 500 μL of Dulbecco’s Modified Eagle Medium (DMEM), ensuring a cement surface area to solution ratio within the International Standards Organization’s recommended range. The 48-well plates containing the disks were incubated at 37°C with daily DMEM replacement. Extracts from the first and seventh days were collected and preserved at -20°C for subsequent cytotoxicity analysis. Human gingival fibroblasts (HGFs) were employed to assess the cytotoxicity of the orthodontic cements. HGFs were cultured in 96-well plates using the extracts supplemented with 100 IU/mL penicillin, 100 IU/mL streptomycin, and 10% fetal calf serum at 37°C for 24 hours. HGFs incubated with a medium devoid of cement extracts served as the control. Cell density was determined using an MTT assay at OD492 nm, and cell viability was inferred from the OD ratios of the experimental groups relative to the control group.

Statistical analysis

Data were processed using IBM Corp. Released 2010. IBM SPSS Statistics for Windows, Version 19.0. Armonk, NY: IBM Corp. The Shapiro-Wilk test was employed to confirm data normality. Variance was examined through one-way and two-way ANOVA, succeeded by Tukey’s multiple comparison test to identify significant differences. A p-value below 0.05 was considered statistically significant.

## Results

**Figure 1:**
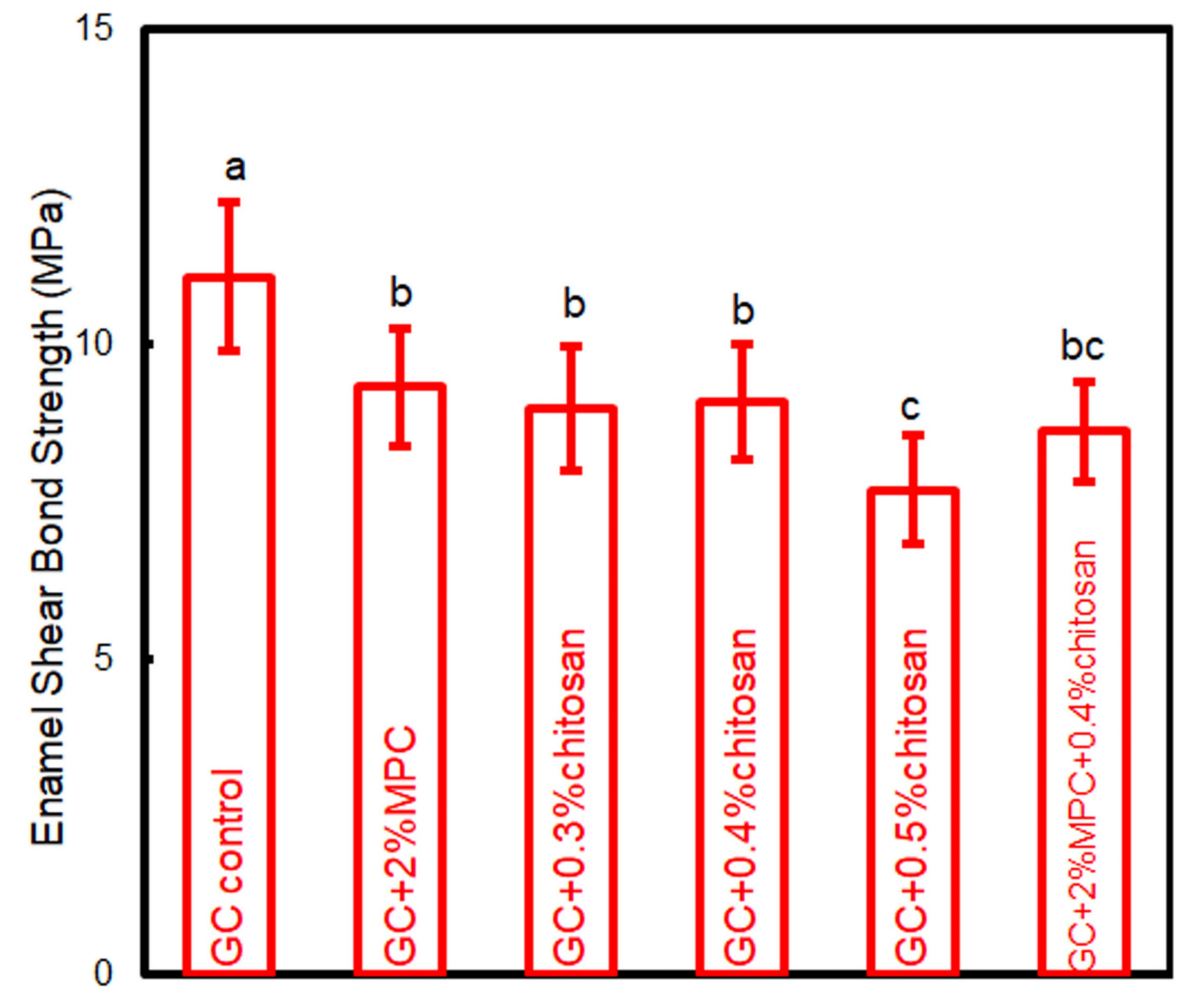
Enamel shear bonding strength of orthodontic cements The same letters indicate no difference between the groups (p > 0.05), while the different letters indicate a significant difference (p < 0.05).

The bonding strength results are shown in Figure 1. An SBS of 7.8 MPa was used as the minimum criterion, according to previous studies [4,18]. GC control exhibited the greatest bonding strength. The incorporation of 2% MPC or chitosan in concentrations of 0.3% and 0.4% resulted in a reduction of SBS compared to the control, albeit remaining above the threshold of 7.8 MPa. An increment in chitosan mass fraction to 0.5% precipitated a further diminution in bonding strength to below the threshold. The addition of 2% MPC and 0.4% chitosan achieved an SBS of 8 61 MPa.

**Figure 2:**
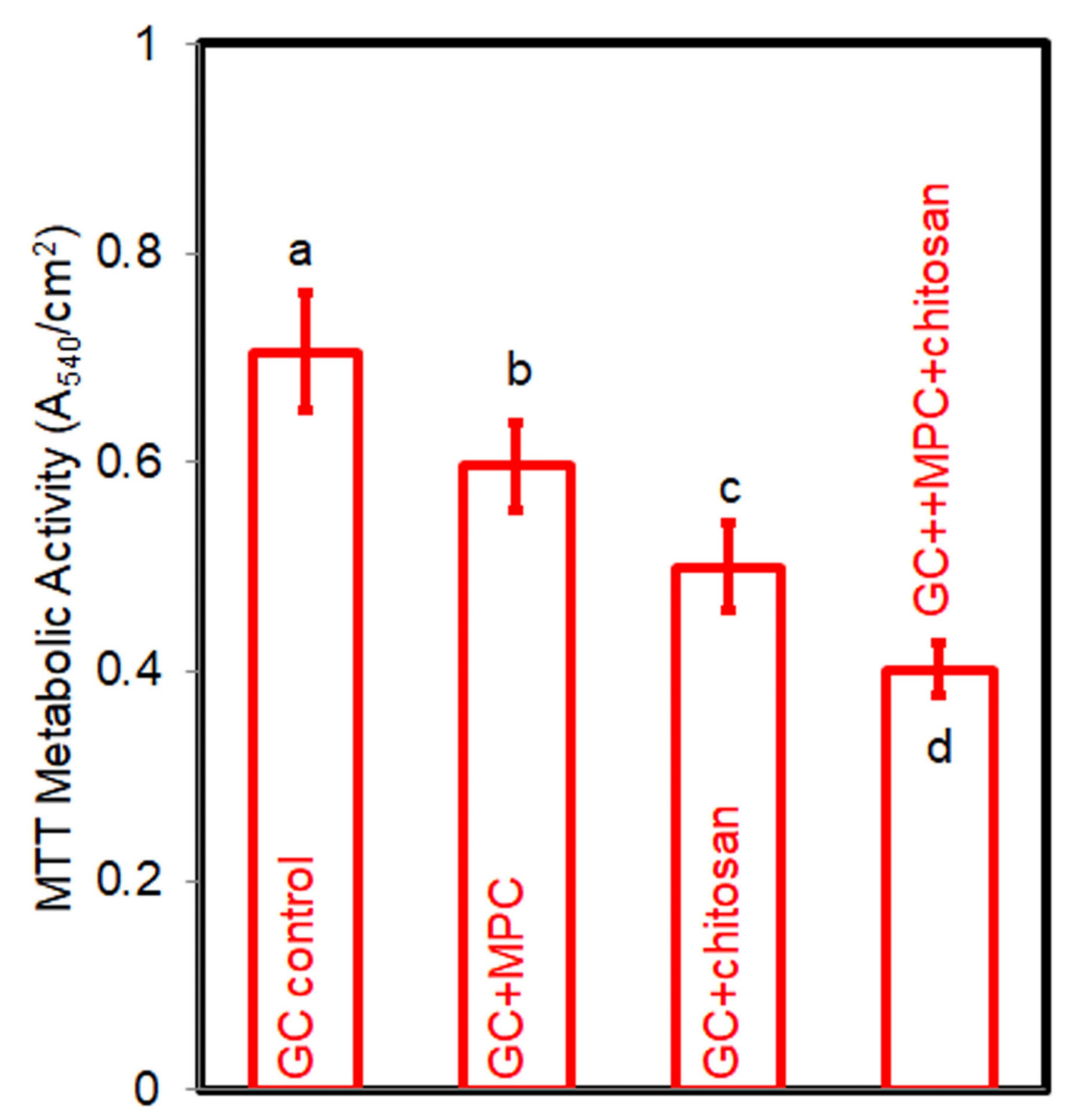
MTT assay of the biofilms on cement disks The same letters indicate no difference between the groups (p > 0.05), while the different letters indicate a significant difference (p < 0.05).

**Figure 3:**
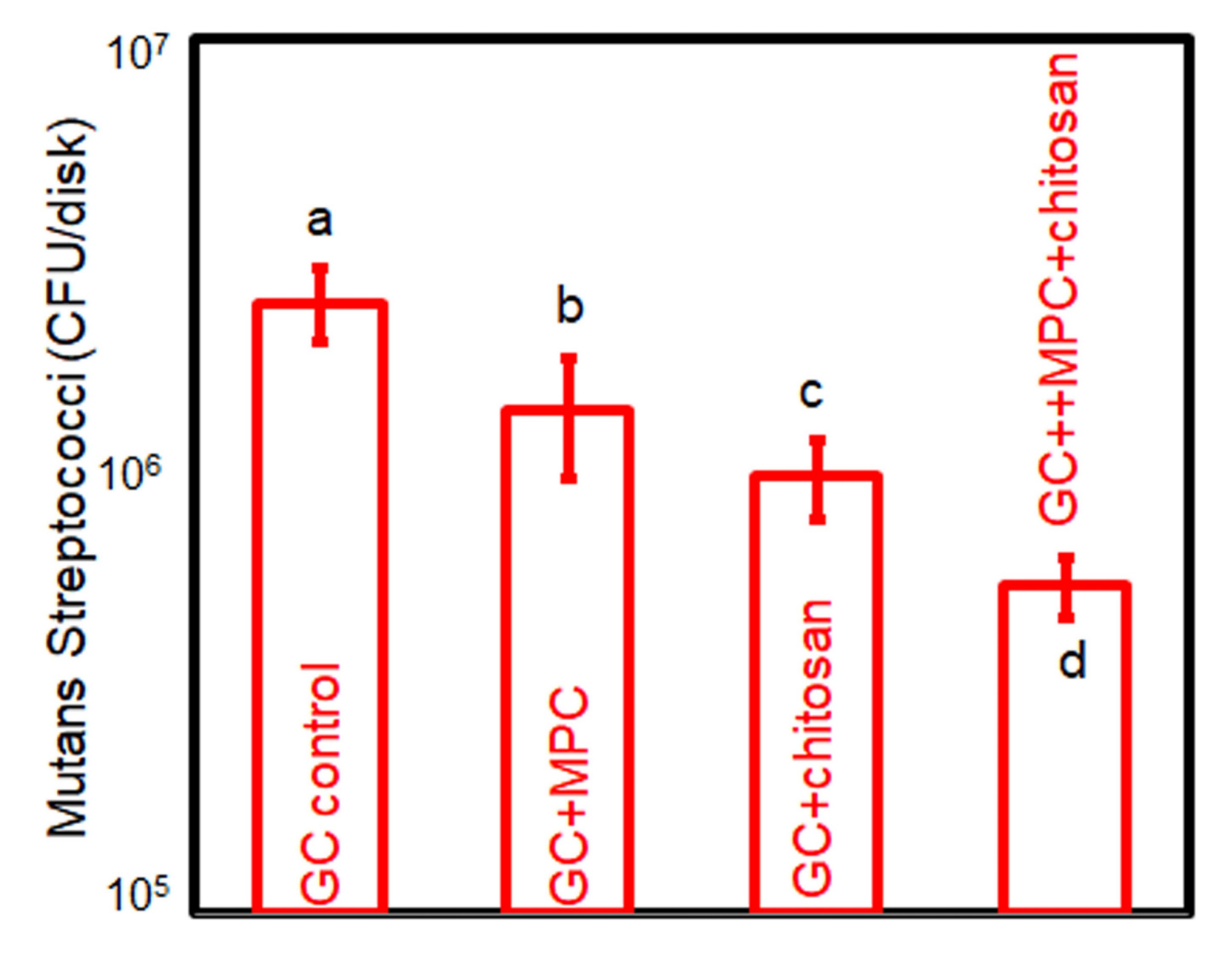
CFU counts of total S. mutans on cement disks The same letters indicate no difference between the groups (p > 0.05), while the different letters indicate a significant difference (p < 0.05).

The metabolic activity results are illustrated in Figure 2. In comparison to the GC control, the metabolic activity of biofilms on cement disks was significantly impeded by the incorporation of MPC (p < 0.05). Chitosan exhibited a more pronounced inhibitory effect on metabolic activity than MPC (p < 0.05). The combined application of both MPC and chitosan yielded the most substantial reduction in metabolic activity (p < 0.05). The CFU counts of two-day biofilms, as depicted in Figure 3, mirrored the metabolic activity trend. The individual utilization of MPC and chitosan effectively curtailed the CFU counts (p < 0.05), while their combined incorporation led to the lowest CFU counts (p < 0.05).

**Figure 4:**
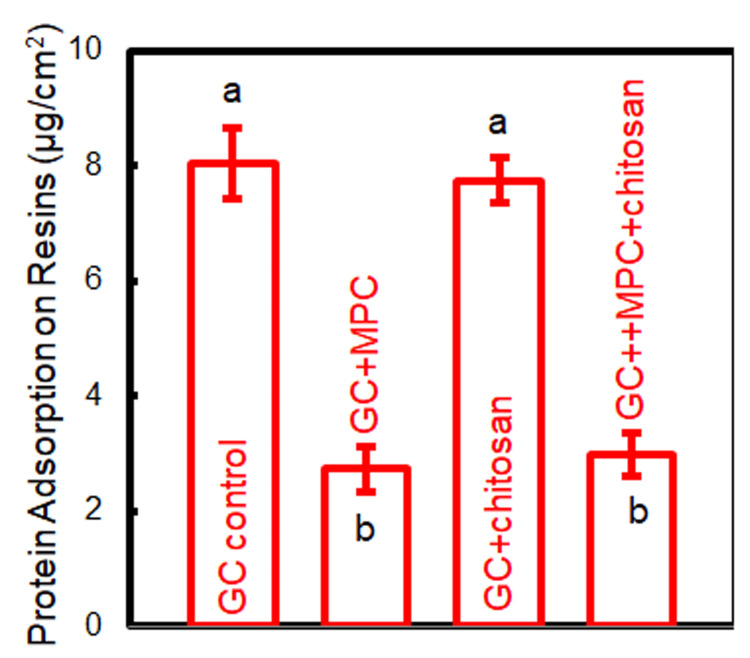
Protein adsorption on cement disks. The same letters indicate no difference between the groups (p > 0.05), while the different letters indicate a significant difference (p < 0.05).

The protein adsorption on cement disks is presented in Figure 4. The addition of MPC greatly attenuated protein adsorption (p < 0.05). The use of chitosan had no obvious effect on the amount of adsorbed protein (p > 0.05).

**Figure 5:**
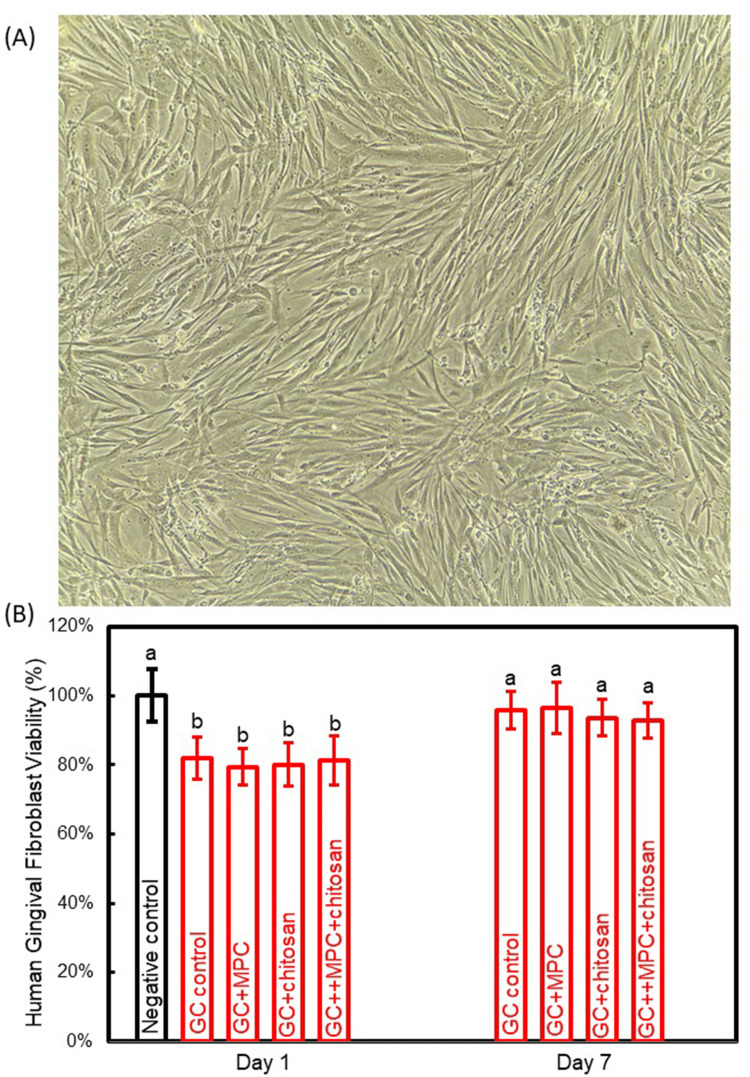
Cytotoxicity of cement disks (A) Microscopic observation of human gingival fibroblasts. (B) Cell viability against cement extracts from days 1 and 7. The same letters indicate no difference between the groups (p > 0.05), while the different letters indicate a significant difference (p < 0.05).

The cytotoxicity evaluation of cements is shown in Figure 5. The viability of HGFs in response to orthodontic cements is used to ascertain cytotoxicity. A cell viability exceeding 70% of the negative control was deemed indicative of acceptable cytotoxicity, in accordance with the International Standard Organization (ISO) guidelines [19]. The addition of MPC and chitosan did not adversely affect cell viability (p > 0.05). The cement containing MPC and chitosan had a cell viability of 81.2% and 92.3% against the extracts of orthodontic cement on days 1 and 7, respectively. 

## Discussion

In this study, a novel orthodontic cement has been formulated by integrating chitosan and MPC into a commercial base to enhance its antibacterial efficacy, resistance to protein adhesion, and biocompatibility. Our findings support that the strategic addition of chitosan and MPC in precise proportions maintains cements’ bonding strength to meet clinical requirements. Furthermore, this novel cement significantly suppresses biofilm activity and growth, reduces protein adhesion, and upholds commendable biocompatibility. 

The bonding strength is a critical parameter for orthodontic cements, necessitating a balance that ensures bracket stability throughout the treatment without impeding bracket removal post-treatment. The integration of bioactive substances into orthodontic cement raises concerns about the potential reduction in adhesive strength. The clinically recognized minimum SBS is 7.8 MPa [18]. It was found that a chitosan concentration exceeding 0.5% resulted in an SBS below this threshold [18]. Consequently, we optimized the chitosan content to 0.4%. The final formulation, comprising 2% MPC and 0.4% chitosan, demonstrated an SBS of 8.61 MPa, thereby qualifying the newly developed cement for clinical use.

The development of WSLs during fixed orthodontic treatment is a direct consequence of biofilm activity, making potent antibacterial properties essential [20]. The cement formulated with MPC and chitosan demonstrated robust antibacterial effects, as evidenced by a 43.1% decrease in biofilm metabolic activity and a reduction of approximately one logarithmic unit in biofilm CFU counts. The mechanisms by which chitosan exerts its antibacterial effects are multifaceted. It involves binding to bacterial cell walls due to its polycationic nature, disrupting the walls and altering membrane permeability, leading to bacterial death [21]. In addition, chitosan chelates metal ions that are essential for bacterial survival, depriving bacteria of the necessary nutrients and inhibiting biofilm growth and reproduction [22]. It also interferes with bacterial DNA, inhibiting DNA replication and cell division [23].

The significant antibacterial property of this novel cement is also attributed to the protein-repelling effect induced by MPC. The cement with MPC shows protein adsorption levels approximately one-third of those without MPC. The phosphorylcholine group of MPC hinders electrostatic interactions with proteins, thus deterring protein adsorption [24]. Its highly hydrophilic nature forms a hydration shell, creating a barrier that repels proteins and prevents their attachment to the cement surface [25]. The combined antibacterial action of chitosan and the protein-repelling capability of MPC synergistically confer exceptional antibacterial properties to the new cement.

The biocompatibility of orthodontic cement is of paramount importance for its application in the oral cavity. The bioactive agents employed in this study, chitosan and MPC, are renowned for their excellent biocompatibility. Chitosan, derived from natural sources, closely resembles glycosaminoglycans in the human body [26]. It is biodegradable, metabolized by lysozyme and other enzymes into non-toxic products, and has demonstrated no adverse effects on animal tissues or cytotoxicity in human fibroblasts [27]. The biocompatibility of MPC, which is likely due to its similarity with the phospholipid bilayer of cell membranes, has also been previously corroborated by in vivo studies [28]. In this study, the cements containing chitosan and MPC showed satisfactory biocompatibility, with cell viability against the extracts from cements surpassing ISO standards, indicating that the concomitant use of chitosan and MPC is a viable and safe strategy to prevent the development of WSLs in clinical settings.

The primary limitation of this study lies in its in vitro design. Despite significant efforts to accurately replicate in vivo conditions, critical factors such as saliva flow were not simulated. Future in vivo studies are essential to validate the efficacy of incorporating chitosan and MPC into orthodontic cement for the prevention of WSLs.

## Conclusions

This study has formulated a novel orthodontic cement that possesses antibacterial properties and meets the criteria for biocompatibility by integrating chitosan and MPC into commercial cement for the first time. The cement achieved clinically acceptable enamel bonding strength. It also demonstrated formidable antibacterial effects, notably impeding biofilm metabolic activity and reducing CFU counts. Moreover, the new cement showed satisfactory biocompatibility. The present study validates the synergistic use of chitosan and MPC as a promising method to shield enamel from demineralization during orthodontic treatment. It also potentially offers a novel strategy for caries prevention.
